# Cell-free DNA metagenomic next generation sequencing for the diagnosis of infectious diseases: a retrospective assessment of clinical utility in a tertiary and quaternary care facility

**DOI:** 10.3389/fmicb.2026.1879762

**Published:** 2026-08-11

**Authors:** Lucas J. Osborn, Apurva Akkad, Neha Nanda

**Affiliations:** 1 Department of Pathology, Keck School of Medicine, University of Southern California, Los Angeles, CA, United States; 2 Department of Medicine, Division of Infectious Disease, Keck School of Medicine, University of Southern California, Los Angeles, CA, United States

**Keywords:** cell-free DNA sequencing, clinical diagnostics, infectious diseases, metagenomics, next generation sequencing

## Abstract

**Background:**

We sought to retrospectively assess the clinical utility of a commercially available plasma cell-free DNA (cfDNA) metagenomic next generation sequencing (mNGS) known as the Karius test (KT), for the diagnosis of infectious diseases, stratified by clinical syndrome.

**Methods:**

Retrospective chart review and abstraction were performed to assess the clinical impact of KT. Descriptive statistics were used to characterize the results of the KT in conjunction with host parameters.

**Results:**

This study included 120 KT results from 114 patients, collected from September 1, 2021, through August 31^st^, 2024, at our academic medical center which includes a 401-bed acute care, academic hospital offering quaternary care, in addition to a 60-bed cancer hospital. The KT demonstrated clinical utility in 28.3% of all 120 cases, with 67.5% yielding no impact, 2.50% negative impact, and 1.67% indeterminate impact, though impact proportions were syndrome dependent. Host immune competency was not a reliable predictor of KT impact, consistent with previous studies. Overall, the KT yielded 100 unique organisms with a 67.5% positivity rate.

**Conclusion:**

Taken together, these data suggest that despite the relatively high rate of organism detection by KT, clinical impact is modest. Based on effect size analyses, this study identified several clinical scenarios that warrant larger scale prospective investigation to more completely elucidate high-yield use cases of KT.

## Introduction

The rapid identification of pathogens in critically ill patients is crucial for guiding antimicrobial therapy and improving patient outcomes. Traditional microbiological methods, including culture-based techniques, remain the gold standard for pathogen detection but are often limited by lengthy turnaround times, low sensitivity in certain clinical contexts, and an inability to detect unculturable or fastidious organisms. These limitations can delay appropriate treatment decisions, contribute to unnecessary antimicrobial use, and increase the risk of antimicrobial resistance.

The clinical application of cfDNA next generation sequencing (NGS) is not unique to infectious disease diagnosis. As early as 2011, cfDNA testing was commercially available for prenatal aneuploidy screening (Grace et al., 2016). Just 5 years later, several professional societies revised their guidelines to recommend cfDNA screening is available for all pregnancies based on the performance of the test for this application and the advantage of a non-invasive “liquid biopsy” (Grace et al., 2016; Parham et al., 2017; Bianchi et al., 2014). Similarly, liquid biopsies have been used to detect circulating tumor DNA for diagnosis, treatment selection, and prognosis in oncologic medicine, including incidental maternal cancer detection during prenatal aneuploidy screening (Devereaux et al., 2023; Domínguez-Vigil et al., 2017; Turriff et al., 2024). Currently, there are multiple FDA-cleared liquid biopsy tests available for the detection of human genomic mutations (U.S. Food and Drug Administration, 2026).

Given the success of cfDNA NGS as prenatal and oncology diagnostics, it’s no surprise cfDNA mNGS has emerged as an increasingly utilized tool for infectious disease diagnosis, offering a culture-independent approach to detecting a broad range of bacterial, viral, fungal, and parasitic pathogens directly from patient plasma (Rossoff et al., 2019; Blauwkamp et al., 2019; Hill et al., 2021; Osborn et al., 2024). Previous studies have demonstrated positive clinical impact for culture negative endocarditis and in the detection of fastidious, zoonotic, and vector borne pathogens (Eichenberger et al., 2022; Kim et al., 2025; Kaur et al., 2025; Khalil et al., 2025). Despite the initial excitement over adaptation of cfDNA mNGS, particularly the KT, for infectious disease diagnosis, the clinical utility is not clearly established (Graf et al., 2025; Niles et al., 2023; Niles et al., 2020; Linder and Miceli, 2023). At the completion of this study, the KT has yet to receive FDA-approval despite its designation as a “breakthrough device.” This designation serves as a mechanism to expedite premarket review, 510(k) clearance, and post-clearance marketing approval for devices addressing an unmet clinical need for more effective diagnosis of life-threatening or irreversibly debilitating diseases and conditions. This voluntary program does not, however, circumvent the rigorous nature of the FDA approval process, nor does it guarantee 510(k) clearance. The KT received breakthrough device designation following the PICKUP study, a multicenter prospective observational study designed to compare KT to the standard of care for diagnosis of pneumonia in adults with hematologic malignancies or previous hematopoietic cell transplantation, where KT was observed to provide 12.1% additive diagnostic value (Bergin et al., 2023). Outside of the specific clinical scenario investigated in the PICKUP study, key questions persist regarding its analytical performance compared to standard-of-care methods, its impact on clinical decision-making, its role in antimicrobial stewardship, and the cost–benefit profile of KT (Kaur et al., 2025; Graf et al., 2025). Current barriers to widespread adoption of KT include cost and reimbursement, interpretive challenges, and reportedly low overall clinical impact. This study aims to assess the real-world utility of KT, an infectious diseases cfDNA mNGS assay, by conducting a single-center retrospective cohort analysis to evaluate its diagnostic yield, concordance with conventional microbiologic testing, and influence on clinical management, and to identify potential clinical scenarios warranting sufficiently powered prospective studies.

## Methods

The study population included patients who had KT collected from September 1, 2021, through August 31st, 2024 at Keck Medicine of USC. Electronic health records were retrospectively reviewed with the following data abstracted for subsequent analysis: demographic data including age; sex and race; medications; diagnostic imaging; clinical history including immune status and signs and symptoms at the time of testing; laboratory values; clinical response; and date of admission/discharge if inpatient. Laboratory values included bacterial, fungal, and mycobacterial culture; infectious diseases antigen and antibody tests; targeted PCR assays, broad-range PCR with reflex to NGS; complete blood counts and differential; acute phase reactants; histopathology reports; and KT (Blauwkamp et al., 2019). Immunocompromise was defined as any immunocompromising condition including immunosuppressive dosing of corticosteroids. If a patient had underlying immunocompromise but a different clinical syndrome was the primary indication for KT, the primary syndrome was categorized in Table 1, while immunocompromised status was captured as “immunocompromised, any” in Table 2. Patients with any history of HSCT or SOT were included in the respective transplant subgrouping, regardless of proximity to KT collection. Patients in the HSCT, SOT, and “immunocompromised, other” groups were combined into a single category, “immunocompromised, combined” for additional statistical comparisons. Clinical impact adjudication was performed with multi-disciplinary representation of three board-certified infectious diseases physicians and a board-certified medical microbiologist. Categorization of clinical impact was based on modified criteria published by Hogan et al. (2021) (Supplementary Table 1). Impact designation for each case was determined through holistic assessment of all abstracted clinical data, including underlying conditions, infection foci, conventional microbiological results, therapeutic changes, and clinical trajectory, consistent with previous studies (Kaur et al., 2025; Hogan et al., 2021; Benoit et al., 2024). Discrepancies between KT and conventional microbiological testing were resolved through consensus informed by the collective clinical experience of the adjudication team. The data used for abstraction is included as Supplementary file 1.

**Table 1:** Clinical characteristics and Karius test utility.

Clinical characteristics	*n* (%)
Sex	
Female	48 (40)
Male	72 (60)
Median age (IQR), years	61.5 (46.5–68.1)
Indication for KT	
Culture negative	86 (71.7)
Evaluate for additional pathogen	24 (20)
Other	10 (8.3)
Syndrome	
SOT	8 (6.7)
HSCT	10 (8.3)
Immunocompromised, other	12 (10.0)
Immunocompromised, any	70 (58.3)
Cardiovascular/Endovascular	37 (30.8)
Bone/Joint	4 (3.3)
Respiratory	20 (16.7)
Neurologic	9 (7.5)
Hemodynamic/Lab abnormalities	3 (2.5)
Gastrointestinal	4 (3.3)
Genitourinary	1 (0.8)
FUO and neutropenic fever	8 (6.7)
Other	4 (3.3)
Median length of stay (IQR), days	20 (13–44)
Clinical impact of KT	
Positive	34 (28.3)
Negative	3 (2.50)
No impact	81 (67.5)
Indeterminate	2 (1.67)

**Table 2:** Statistical analysis with complementary composite as reference comparison.

Syndrome	*N*	Complement reference *n*	Impact *n* [positive, negative, none, indeterminate]	Δ vs reference^†^ [positive, negative, none, indeterminate]	*χ* ^2^	*p*-value	Cohen *ω*
Composite reference	120	—	[34, 3, 81, 2]	—	—	—	—
Immunocompetent	50	70	[14, 2, 32, 2]	[−0.6%, +2.6%, −6.0%, +4.0%]	3.730	0.292	0.256
Immunocompromised, any	70	50	[20, 1, 49, 0]	[+0.6%, −2.6%, +6.0%, −4.0%]	3.730	0.292	0.256
↳Immunocompromised, combined^††^	30	90	[10, 0, 20, 0]	[+6.7%, −3.3%, −1.1%, −2.2%]	2.024	0.568	0.237
↳Immunocompromised, combined^*^	30	50	[10, 0, 20, 0]	[+5.3%, −4.0%, +2.7%, −4.0%]	2.598	0.458	0.271
↳Immunocompromised, other	12	108	[3, 0, 9, 0]	[−3.7%, −2.8%, +8.3%, −1.9%]	0.719	0.869	0.234
↳Immunocompromised, SOT	8	112	[5, 0, 3, 0]	[+36.6%, −2.7%, −32.1%, −1.8%]	5.032	0.170	0.768
↳Immunocompromised, HSCT	10	110	[2, 0, 8, 0]	[−9.1%, −2.7%, +13.6%, −1.8%]	0.975	0.807	0.300
Cardiovascular/Endovascular	37	83	[10, 1, 25, 1]	[−1.9%, +0.3%, +0.1%, +1.5%]	0.386	0.943	0.093
↳Endocarditis^**^	24	96	[5, 1, 17, 1]	[−9.4%, +2.1%, +4.2%, +3.1%]	2.103	0.551	0.273
Bone/Joint	4	116	[1, 0, 3, 0]	[−3.4%, −2.6%, +7.8%, −1.7%]	—	1.000	—
Respiratory	20	100	[6, 1, 13, 0]	[+2.0%, +3.0%, −3.0%, −2.0%]	1.046	0.790	0.213
Neurologic	9	111	[1, 0, 8, 0]	[−18.6%, −2.7%, +23.1%, −1.8%]	2.083	0.555	0.464
Hemodynamic/Lab abnormalities	3	117	[0, 1, 2, 0]	[−29.1%, +31.6%, −0.9%, −1.7%]	—	0.558	—
Gastrointestinal	4	116	[2, 0, 2, 0]	[+22.4%, −2.6%, −18.1%, −1.7%]	—	0.318	—
Other	4	116	[0, 0, 3, 1]	[−29.3%, −2.6%, +7.8%, +24.1%]	—	0.576	—
Genitourinary	1	119	[0, 0, 1, 0]	[−28.6%, −2.5%, +32.8%, −1.7%]	—	1.000	—
FUO and neutropenic fever	8	112	[4, 0, 4, 0]	[+23.2%, −2.7%, −18.8%, −1.8%]	2.166	0.539	0.504

Chi-square (2 × 4) across positive, negative, no impact, indeterminate categories. Fisher’s exact applied as 2 × 2 (positive vs. all others) for groups with *n* < 5. ^†^Composite reference minus subgroup complement. ^††^Includes SOT; HSCT; and immunocompromised, other groups. ^*^Statistical comparison with immunocompetent as reference group. ^**^Subgroup comprised of encounters 8, 14, 19, 20, 27, 28, 34, 35, 37, 41, 59, 61, 64, 74, 75, 81, 84, 91, 92, 98, 101, 115, 116, 120 (Supplementary file 1).

### Statistical analysis

Statistical comparisons were performed to assess whether distributions of outcomes (positive impact, negative impact, no impact, and indeterminate) differed significantly across subgroups. For subgroups with *n* ≥ 5, Pearson’s chi-square tests of independence were applied to 2 × 4 contingency tables encompassing all four outcome categories. For subgroups with *n* < 5 (bone/joint, hemodynamic/lab abnormalities, gastrointestinal, genitourinary, and other), Fisher’s exact test was applied to 2 × 2 contingency tables in which outcomes were collapsed into positive impact versus all other categories combined, consistent with the constraints of the Fisher’s exact method. Each subgroup was compared against its complement, i.e., the remaining subjects in the reference after excluding that subgroup. The directionality of effect was determined by calculating the Δ% deviation from the complement reference group for all four impact categories. Effect size was calculated using Cohen’s *ω* where conventional effect sizes were applied: small ≥0.1–<0.3, moderate 0.3–<0.5, large ≥0.5 (Cohen, 1988). A significance threshold of *α* = 0.05 was applied to all comparisons without correction for multiple testing, consistent with the exploratory nature of this study. All analyses were performed using a Python (version 3.9.14) script generated by Claude for Mac (version 1.3883.0), executed in Spyder IDE (version 5.5.2) with the SciPy statistical library.

## Results

### Patient characteristics and KT results

A total of 120 patient encounters were included (40% female), with a median age of 61.5 years (IQR 46.5–68.1). The most common indications for testing were culture-negative clinical scenarios (71.7%), followed by evaluation for additional pathogens (20%) (Table 1). Over half of patients were immunocompromised (58.3%), including solid organ transplant (SOT), hematopoietic stem cell transplant (HSCT), and other causes (Table 1; Supplementary file 1). The positivity rate of KT was 67.5% with 100 unique organisms identified, predominantly bacteria (46.4%) (Supplementary Figures 1A–C; Supplementary file 1). Most KT reports identified between 0–2 organisms, though several reports listed more than 5 organisms with one report listing 9 taxa (Supplementary Figure 1B; Supplementary file 1). The ICD-10 code descriptors most frequently associated with KT orders were myeloid leukemia (10.4%), other sepsis (7.97%), respiratory failure (7.42%), and endocarditis (4.67%) (Supplementary Figure 1D).

### Utility of KT by syndrome

**Figure 1:**
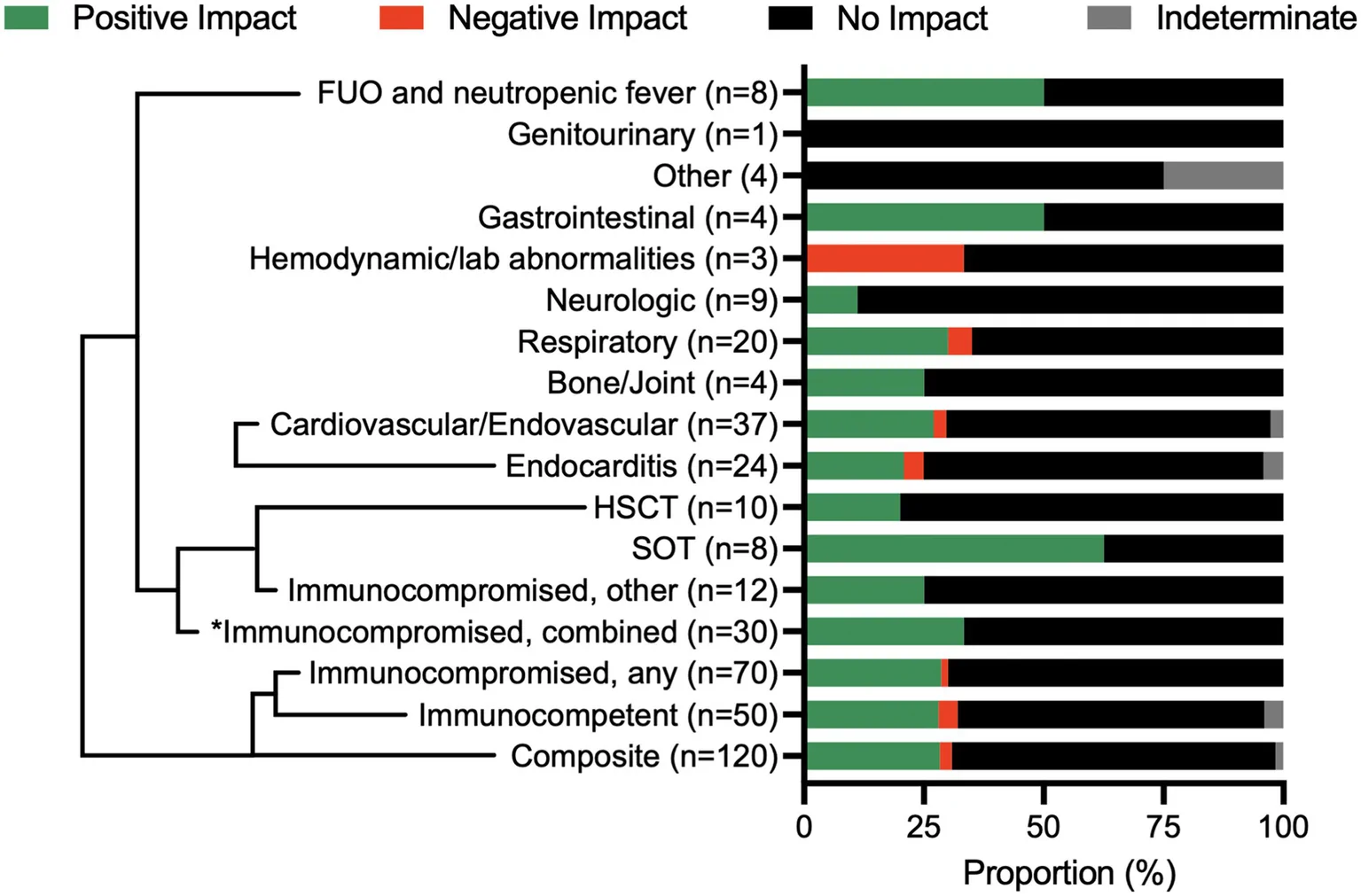
Impact of Karius test by syndrome. The clinical impact of KT was determined by retrospective chart abstraction and stratified by syndrome. Solid black lines represent the aggregation of subgroups. *Includes SOT; HSCT; and immunocompromised, other groups.

Among immunocompromised patients, clinical impact varied by subgroup but were overall comparable to the composite cohort (Figure 1; Table 2). In solid organ transplant recipients (*n* = 8), positive clinical impact was observed in 5 (62.5%) with no impact in the remaining 3 (37.5%) patients, representing a large effect size (Cohen’s *ω* = 0.768) with 36.6% increased positive utility over the complement reference. In contrast, a lower rate of positive clinical impact was observed in hematopoietic stem cell transplant recipients (*n* = 10), where 2 (20%) KT results had a positive clinical impact and 8 (80%) had no impact. Similar results were observed among patients with other non-transplant related immunocompromising conditions (*n* = 12) where the majority (75%) of KT results had no clinical impact. When analyzed collectively, the clinical impact of KT for patients with any immunocompromising condition (*n* = 70) or those in the combined immunocompromise group (HSCT, SOT, and other immunocompromised) (*n* = 30) did not significantly differ from immunocompetent patients (*p* = 0.292 and *p* = 0.458, respectively) (Table 2).

Among patients with cardiovascular or endovascular syndromes (*n* = 37), positive clinical impact was observed in 10 (27.0%), no impact in 25 (67.6%), and one case of both indeterminate and negative impacts (2.7%). Of the 37 patients in the cardiovascular or endovascular group, 24 were specifically evaluated for endocarditis. Further subgroup analysis of these 24 patients revealed organism detection by KT in 50% (*n* = 12). Two of the positive KT results established a new pathogen for diagnosis not otherwise confirmed by conventional microbiological testing (Supplementary file 1). In one case, KT identified *Coxiella burnetii* 4 days prior to serology and PCR thus representing a diagnostic time advantage. However, the majority of the positive KT results for endocarditis were not clinically actionable (*n* = 7). Notably, *Cutibacterium acnes* was identified by valve tissue culture and PCR despite negative KT, illustrating a false negative in the endocarditis sub cohort. For patients with respiratory syndromes (*n* = 20), clinical impact was positive in 6 (30%), negative in 1 (5%), and absent in 13 (65%). Clinical impact of KT was less frequent in patients with neurologic syndromes (*n* = 9) where a single case (11.1%) resulted in positive impact whereas the remaining 8 (88.9%) had no impact with an observed −18.6 and +23.1% deviation from positive and no impact categories in the complement reference, respectively and a moderate effect size (Cohen’s *ω* = 0.464). Among patients with fever of unknown origin or neutropenic fever (*n* = 8), clinical impact was equally divided between positive clinical impact (50%) and no impact (50%); representing a +23.2% and −18.8% deviation from positive and no impact categories in the complement reference, respectively with a large effect size (Cohen’s *ω* = 0.504).

Syndromes with small sample sizes included bone and joint infections (*n* = 4), gastrointestinal (GI) and genitourinary (GU) syndromes (*n* = 5), and hemodynamic or laboratory abnormalities (*n* = 3). Among these groups, positive clinical impact was observed in 1 of 4 (25%) bone and joint cases, 2 of 5 (40%) GI/GU cases, and 0 of 3 hemodynamic or laboratory abnormality cases. The KT resulted in no clinical impact for the remaining small sample size cohort apart from negative clinical impact observed in 1 of 3 (33.3%) patients with hemodynamic or laboratory abnormalities. Overall, negative clinical impact was uncommon, occurring in 3 cases (2.5%) where KT results led to additional investigations that were later deemed unnecessary.

## Discussion

Here we report the clinical utility of KT in our tertiary and quaternary care academic medical center. Overall, 28.3% of KT included in this study yielded a positive clinical impact. This proportion is slightly higher than other studies, perhaps explained in-part by the requirement for an infectious diseases physician to approve KT requests at our facility (Kaur et al., 2025; Graf et al., 2025; Niles et al., 2023; Niles et al., 2020; Lee et al., 2020; Hogan et al., 2021). Consistent with the literature, host immunocompromised status was not significantly associated with a positive clinical impact of KT in our study cohort (Kaur et al., 2025; Lee et al., 2020). Based on our analysis of KT impact by clinical presentation, none of the syndromic categories benefited from improved KT impact compared to the complementary reference cohort. Despite this observation, the analytical performance of cfDNA mNGS is reportedly dependent on the indication for testing. The literature to date suggests culture-negative endocarditis in the setting of prolonged antecedent antimicrobial therapy is one of the highest-yield clinical indications for KT (Eichenberger et al., 2022; Khalil et al., 2025). These findings remain unsurprising given that plasma is directly contiguous with the source of infection in patients with endocarditis. However, several studies have elucidated notable false negative and false positive KT results in the setting of culture-negative endocarditis, suggesting careful interpretation of negative results is still warranted and underscored by a false negative KT for *Cutibacterium acnes* endocarditis in this study (Eichenberger et al., 2022; Khalil et al., 2025). While biologically plausible as a potentially high yield indication for KT, our analysis suggests a more mixed clinical impact with additive diagnostic value realized in a minority of cases and a substantial proportion of results that were not clinically actionable.

Across the literature, mNGS shows variable utility in hospitalized patients with underlying hematologic malignancies presenting with febrile neutropenia (FN) (Brown et al., 2026). Feng et al. (Feng et al., 2024) reported positive and negative percent agreement of 81.9 and 60.9%, respectively, with treatment modification in 36% of cases. Schulz et al. (Schulz et al., 2022) report a 2-fold higher sensitivity and broader pathogen spectrum by mNGS compared to blood cultures in FN patients. However, important limitations were noted including relatively low sensitivity for bacteria (40% [95% CI, 28.0–52.9%]) and fungi (18.5% [95% CI, 9.9–30.0%]), as well as approximately 15% false-negative mNGS results in cases with positive blood cultures, indicating that mNGS should not be considered a rule-out test. Consistent with these limitations, Benamu et al. reported notable false negative mNGS results in FN patients (Benamu et al., 2022). In the current study, the combined FN and fever of unknown origin (FUO) subgroup revealed a moderate effect size (Cohen’s *ω* = 0.504) with 50% of cases classified as positive impact compared to 27% in the complementary cohort. These data may suggest a FN/FUO as a potentially high yield scenario as was described in a recent retrospective study evaluating the utility of KT for FUO and a small prospective study in patients with FN (Benamu et al., 2022; Ranganath et al., 2025). Although statistical significance was not observed for FN/FUO in this study, these findings may be explained in part by the afore mentioned sensitivity limitations and/or the relatively small sample size our study subpopulation (*n* = 8).

Outside of febrile neutropenia, the additive diagnostic value of mNGS appears inconsistent and often limited (Huygens et al., 2024). In immunocompromised patients with pneumonia, one study reported only a 12.1% additive diagnostic yield, with frequent discordance compared to conventional testing (Bergin et al., 2023), while studies in abdominal infections suggest modest utility with concerns about detection of clinically irrelevant organisms (Kaur et al., 2025; Niles et al., 2023; Lee et al., 2020; Han et al., 2023). In neurologic infections, plasma mNGS performs poorly relative to cerebrospinal fluid testing, possibly due to infection compartmentalization (Kaur et al., 2025; Linder and Miceli, 2023; Benoit et al., 2024; Chiu and Miller, 2019; Wilson et al., 2019). Evidence in transplant populations remains heterogeneous: retrospective data in solid organ transplant recipients show a positive clinical impact in roughly one in four cases (Shah et al., 2024), while studies in hematopoietic stem cell transplant recipients demonstrate higher organism detection rates but variable concordance and relatively low specificity (Huang et al., 2023; Fu et al., 2024; Liu et al., 2021). In this retrospective cohort study, KT utility was highest in SOT patients with a large effect size (Cohen’s *ω* = 0.768) and 36.6% increased positive utility over the complement reference. The HSCT population in this study revealed −9.1% positive utility compared to the complement reference, with a moderate effect size (Cohen’s *ω* = 0.3). Although these observations did not reach statistical significance, the relatively small sample sizes (*n* = 8 and *n* = 10, respectively) may be insufficiently powered to detect a true difference. Overall, while mNGS may complement existing diagnostics, its clinical utility remains context-dependent, with important limitations in sensitivity, specificity, and interpretation. One important limitation of literature comparison of mNGS utility is that performance metrics are not generalizable amongst different mNGS assays as there is marked variability in preanalytical, analytical, and postanalytical factors including nucleic acid extraction efficiency, computational versus physical removal of host-DNA, sequencing methodology, and bioinformatic processing. Furthermore, a limitation of all studies evaluating the performance of any novel diagnostic, including the data presented herein, is the inherent subjectivity of experience-guided adjudication and impact determination. Additionally, the inclusion of KT results in the final diagnosis carries the risk of introducing circular reasoning. However, the acknowledged subjectivity is largely unavoidable when evaluating the clinical impact of diagnostic testing in complex, real-world patient encounters where no single data element can fully capture the nuance of clinical decision-making.

There are numerous infectious diseases for which targeted PCR-based diagnostics demonstrate superior sensitivity to conventional microbiologic methodologies (Hasan et al., 2025; Caliendo et al., 2013). However, the performance characteristics of PCR-based diagnostics are not broadly applicable to all molecular methodologies such as mNGS. PCR-based diagnostics generally offer a sensitivity advantage over mNGS as a result of amplification following nucleic acid extraction. Conversely, the sensitivity of mNGS largely depends on the quantity of nucleic acids present in the specimen at the time of collection, efficiency of the extraction protocol, and physical or bioinformatic removal of host DNA or host reads, respectively. In our learned experience, the analytical performance of KT is commonly conflated with that of PCR-based molecular diagnostics by non-infectious diseases clinicians. Consequently, subject matter expert interpretation of KT results directly communicated with the patient’s primary physician may aid in conveying the strengths and limitations of a particular assay, ensuring the final interpretation of the results are clinically and diagnostically appropriate. The need for subject matter expert interpretation is underscored by a systematic review and meta-analysis of plasma cfDNA mNGS for infectious disease diagnosis revealed pooled positive and negative likelihood ratios of 2.16 (1.48–3.15, 95% CI) and 0.24 (0.11–0.51, 95% CI) (Niles et al., 2023). Modest performance metrics in this range for other diagnostics have been described by Spellberg et al. (Spellberg et al., 2025) as requiring “…*Bayesian statistical inference at the bedside, which is rarely how many clinicians operate*”.

In our study, a negative impact was noted in 2.5% (3/120) of the cases. In all three cases, KT was used unnecessarily and resulted in additional investigations that were not required. The relatively low proportion of KT leading to negative impact may be explained in part by our institutional diagnostic stewardship requirement for approval by an infectious diseases physician and is consistent with proportion of negative impact cases from previously published studies evaluating the performance of KT with similar approaches to diagnostic stewardship (Kaur et al., 2025; Hogan et al., 2021). To support the philosophy of diagnostic stewardship, several academic institutions across US have adopted the policy of a mandatory infectious disease consultation prior to requesting mNGS testing (Shean et al., 2024; Naureckas Li and Nakamura, 2022). Additionally, as subject matter experts well versed in the analytical performance and limitations of mNGS, the medical microbiologist should be involved in communicating mNGS results directly with the clinical team, to further enhance the interpretation of KT results (Costales and Dien Bard, 2024).

In summary, this retrospective analysis of the clinical utility of KT at an academic medical center offering tertiary and quaternary care with a case mix index of 3.13 at the time of publication (one of the highest in the country), revealed modest positive impact, consistent with other previously published studies (Case Mix Index, 2026; Centers for Medicare and Medicaid Services (CMS), 2026). While our analysis of various clinical syndromes did not yield statistically improved clinical utility when compared to the respective complementary reference, these observations may reflect type II error inflation due to the relatively limited size of this single-center cohort. However, our effect size analysis has identified solid organ transplant, febrile neutropenia, and fever of unknown origin as potentially high-yield scenarios where future well-powered prospective studies are warranted to more definitively understand the performance of KT in various clinical settings, while also reinforcing the low clinical impact of KT for neurologic indications reported by others.

## Data Availability

The original contributions presented in the study are included in the article/Supplementary material, further inquiries can be directed to the corresponding author.

## Supplementary material

Supplementary Figure 1 Details of Karius test orders and results. **(A)** Positivity rate of KT included in this study. **(B)** Number of unique organisms listed in the KT report. **(C)** Categorization of organism or organism class detected by KT. All unique taxa with a prevalence <2.86% of all organisms was categorized as “Other” bacteria, virus, or fungi, as appropriate, for visualization purposes. **(D)** Top 7 most common ICD-10 codes associated with KT orders. All individual ICD-10 codes with prevalence <2.75% were categorized as “Other” for visualization purposes.
